# Trends and determinants of gastric bacterial colonization of preterm neonates in a NICU setting

**DOI:** 10.1371/journal.pone.0114664

**Published:** 2015-07-01

**Authors:** Ketki Patel, Kavitha Konduru, Alok K. Patra, Dinesh S. Chandel, Pinaki Panigrahi

**Affiliations:** 1 Department of Epidemiology, University of Nebraska Medical Center, College of Public Health, Omaha, NE, United States of America; 2 Department of Pediatrics, University of Maryland School of Medicine, Baltimore, MD, United States of America; 3 Department of Environmental, Agricultural and Occupational Health, University of Nebraska Medical Center, College of Public Health, Omaha, NE, United States of America; 4 Department of Pediatrics, University of Nebraska Medical Center, College of Public Health, Omaha, NE, United States of America; 5 Center for Global Health and Development, University of Nebraska Medical Center, College of Public Health, Omaha, NE, United States of America; The Ohio State Unversity, UNITED STATES

## Abstract

**Background:**

Newborn gastrointestinal (GI) tract is considered sterile but rapidly acquires a diverse microbiota from its intimate environment. Early acquisition of a bacterial species in the upper GI tract may play a role in establishing the colonic microbiota. There is paucity of molecular data on the upper GI tract microbiota in preterm neonates.

**Methods:**

Gastric aspirates from 22 neonates with an average gestational age 27.7 weeks (±2.8), weighing 973.2 grams (±297.9) admitted to a neonatal intensive care unit were collected prospectively from weeks 1-4 of life. All samples were evaluated for microbiota using 16S rRNA-based Denaturing Gradient Gel Electrophoresis. Bacterial species colonization and its association with maternal and neonatal demographics, and neonatal clinical characteristics were analyzed.

**Results:**

*Bacteroides spp.* was the predominant species in all four weeks. *Bifidobacterium spp.* colonization was significantly higher in exclusively breast milk fed compared to partially breast milk (PBM) fed neonates in first (p = 0.03) and third (p = 0.03) week of life. Anaerobic bacteria colonization decreased from first through fourth week of life (p = 0.03). Aerobic bacteria colonization was highly dynamic throughout the four week period. Premature rupture of membrane (p = 0.05) and birth outside of study hospital (p = 0.006) influenced the acquisition of bacteria in the first week of life. Birth weight was positively correlated with total number of bacterial species (p = 0.002) and anaerobes (p = 0.004) in PBM-fed neonates during the fourth week of life. *H. pylori* and *Ureaplasma* were not detected in any of our samples.

**Conclusion:**

Gastric bacterial colonization in preterm neonates is unstable during early weeks of life. Delayed oral feeding and use of antibiotics may be responsible for paucity of bacterial species. Monitoring of the gastric microbiota and concurrent examination of stool microbiota may yield important information on the utility of gastric signature patterns for predicting colon microbiota that may drive GI and immune dysfunctions.

## Introduction

During the first two weeks in life, healthy full term neonates get colonized with a diverse and heterogeneous community of bacterial species, with Bifidobacteria predominating in breast-fed infants and a more mixed microbiota in those fed formula. The diversity and heterogeneity of bacterial species continues to grow over the period of first 2–3 years of life [1]. In contrast, the gastrointestinal tract of preterm neonates, cared for in the relatively aseptic neonatal intensive care unit (NICU) environment, usually receiving antibiotic treatment shortly after birth, show delayed colonization with a limited number of bacterial species [2–4]. Preterm neonates due to delayed and abnormal patterns of gastrointestinal colonization, are more susceptible to colonization by potentially pathogenic bacteria [1,4–6]. Even bacterial species considered normal for healthy infants can trigger inflammatory response and may be responsible, in part, for triggering disease such as (NEC) necrotizing enterocolitis [7,8].

Studies traditionally relying on culture techniques have shown over a dozen species in human stool by the end of first week of life [5]. Recent studies using molecular techniques demonstrate that over 80% of the human intestinal tract bacteria are not detected by culture [9,10]. This suggests that the gastrointestinal tract microbial community still remains only partially explored. A large body of literature in adult population has now shown several hundred species form the colon microbiota, many of which still remain unidentified. In the context of newborn microbiota, majority of the culture based studies of the colonic microbiota have focused on term infants [1,11–13]. A handful of studies in preterm infants have demonstrated stool and duodenal bacterial diversity, and possible links between particular bacterial groups such as the enterobacteriaceae and NEC [14,15]. Recent molecular studies on stool microbiota during infancy suggest heterogeneity in bacterial profiles in early weeks of life [3,16].

Although increasing numbers of studies characterize distal gastrointestinal tract microbiota, there is paucity of information on the microbial milieu of the human upper gastrointestinal (GI) tract including the stomach, a primary exposure site (after the oral cavity) to the extraneous bacterial world at birth. Investigators have not emphasized on studies describing acquisition of normal microbiota in upper GI tract of newborns. Although the stomach environment was considered to be sterile (due to low pH), identification of *Helicobacter pylori* in the stomach not only revolutionized the field of gastrointestinal tract microbiology, but also claimed a Nobel prize in medicine due to establishment of its link with peptic ulcer disease. In spite of substantial work being done on colonic microbiota, and attempts made to link bacterial colonization pattern with diseases spanning from NEC in newborns to inflammatory bowel disease in children and adults [17–21], newer molecular techniques have not been extensively applied to study the upper GI microbiota. It is possible that apart from acquisition of bacteria with a potential to cause disease, the so called normal microbiota that first enters the GI tract may ultimately shape the colonization pattern of lower GI tract [8,22,23]. The current study was designed to examine the upper gastrointestinal microbiota during early neonatal development using a 16s rRNA-based denaturing gradient gel electrophoresis (DGGE) approach. Knowledge obtained from such studies, together with information available on lower intestinal colonization may elucidate the role of upper intestinal bacteria in health and disease, and help scientists design targeted interventions and timing of such therapy for specific age groups.

## Materials and Methods

### Study design and population

A prospective cohort of very low birth weight (VLBW) neonates admitted to level III Neonatal Intensive Care Unit (NICU) at University of Maryland Medical System (UMMS) during August 2008- December 2009 were enrolled in the study. The study protocol was approved by the institutional review board at University of Maryland. Written parental consent was obtained prior to enrollment of infants in the study. Parents of eligible neonates were provided information on the description and aim of the study and team’s contact information. Parents had a choice to withdraw from the study at any given stage without any influence on medical care of the neonate. All infants weighing <1500 g, born at UMMS-NICU and those transferred to UMMS-NICU within 24 hours of life were eligible for the study. A total of 72 infants were enrolled in this study. Gastric aspirates from 22 neonates weighing <1500 g at birth were collected weekly (day 7±1, day 14±2, day 21±2, day 28±3) up to 4 weeks, and were subjected to DGGE analyses. In general, infants with gestational age 24–26 weeks, 26–28 weeks, and 28–32 weeks, were fed every 6–8, 4–6, and 4 hourly respectively. Gastric aspiration was done before each feeding implying that the aspirate sat in the stomach for at least four hours before being collected. New feeding was withheld in case of more than small residuals and based on clinical evaluation. Feeding was always withheld if the residual was more than the amount of previous feed. Infant parameters such as birth weight, gestational age, sex, respiratory distress syndrome (RDS), chronic lung disease (CLD), duration of antibiotic use, use of other medications such as H2-blockers, postnatal steroids and information on feeding type (exclusively breast milk (EBM) vs. partially breast milk and preterm formula feed (PBM) were recorded prospectively. PBM included breast milk and formula. Information on maternal parameters such as duration of rupture of membranes, placental pathology, maternal medication use (intra-partum antibiotics), vaginal group B streptococcus colonization, and antenatal use of steroid was obtained. Timing of initial feedings was at the discretion of the attending physician beginning with 20 cc/kg of either breast milk or preterm formula and advanced by 20cc/kg/day until full feeds (150cc/kg/day) were attained. In the first week, all infants were treated with ampicillin and gentamicin or cefotaxime for presumed or confirmed sepsis. Empiric treatment for late onset sepsis (i.e. >72 hours of life) following the first week included vancomycin and cefotaxime. For positive blood cultures, antibiotics were tailored to the sensitivity of the organism. Eleven out of 22 infants in our study population were extremely low birth weight (ELBW) infants (<1000g), and were placed on fungal prophylaxis starting from day 1–3 per the NICU protocol.

### Sample collection, DNA extraction and purification

Gastric aspirates (GA) were collected during routine nursing care and were immediately frozen at -20°C in the NICU. Samples were daily transferred to the laboratory on ice and stored at -70°C and were processed in batches for Denaturing Gradient Gel Electrophoresis (DGGE). Uniform aliquots (250 μl) of aspirates were subjected to genomic DNA extraction using the QIAamp DNA stool kit (Qiagen Inc. Maryland, USA). To a 250 μl sample in a 2 ml tube, 1.4 ml of ASL buffer was added and mixed by vortexing. The resulting homogenate was incubated at 70°C for 10 minutes. To this, 250 mg of Zirconia beads were added and vortexed vigorously for 5–6 minutes, and placed over ice immediately. After centrifugation at maximum speed for 5 minutes, 1.2 ml of supernatant was transferred to a fresh 2 ml tube preloaded with inhibit-Ex tablet, homogenized by vortexing and incubated at room temperature for 5 minutes (PCR inhibitors in samples are adsorbed due to action of Inhibit Ex tablets). The resulting suspension was centrifuged at max-speed for 10 minutes to separate the tablet-inhibitor-complexes from partially purified bacteria-DNA in the supernatant. About 400 μl of the supernatant was transferred to a new 1.5 ml tube and processed in an automated DNA elution Robotic system for the final steps in DNA purification (QIAcube, Qiagen Inc. Maryland, USA).

### PCR amplification of 16S rDNA

The genomic grade DNA purified from gastric samples was subjected to universal PCR targeting V3 region of 16S rRNA gene of bacterial genome using DGGE specific primer pairs [24], where the forward primer was tagged to a 40 base pair (bp) GC-clamp. These primers co-amplify the V3 region (191 bp) of the small subunit ribosomal gene common to all bacterial species resulting in identical sized PCR-products thereby helping in sequence based identification of DNA fragments resolved in a denaturing gradient gel environment in DGGE.

The DGGE PCR conditions, following an initial denaturation at 94°C for 5 minutes, included 35 rounds of cyclic amplification: a denaturing step of 94°C for 30 seconds, primer annealing at 55°C for 30 seconds, extension at 72°C for 1 minute, with an overall final extension for 7 minutes. A 50 μl standard reaction in a 1x PCR buffer (Promega corp. WI, USA) comprised of 1.5 mM MgCl_2_, 200 μM dNTP-mix, 25 pM of each primer (D3 and D5), 2U of Taq DNA-polymerase and 0.5% of formaldehyde to facilitate effective annealing of the GC clamped primer sequences. For a 50 μl reaction, 2 μl of the purified DNA was added to a 48 μl PCR reaction mix.

### Denaturing Gradient Gel Electrophoresis (DGGE)

**Fig 1:**
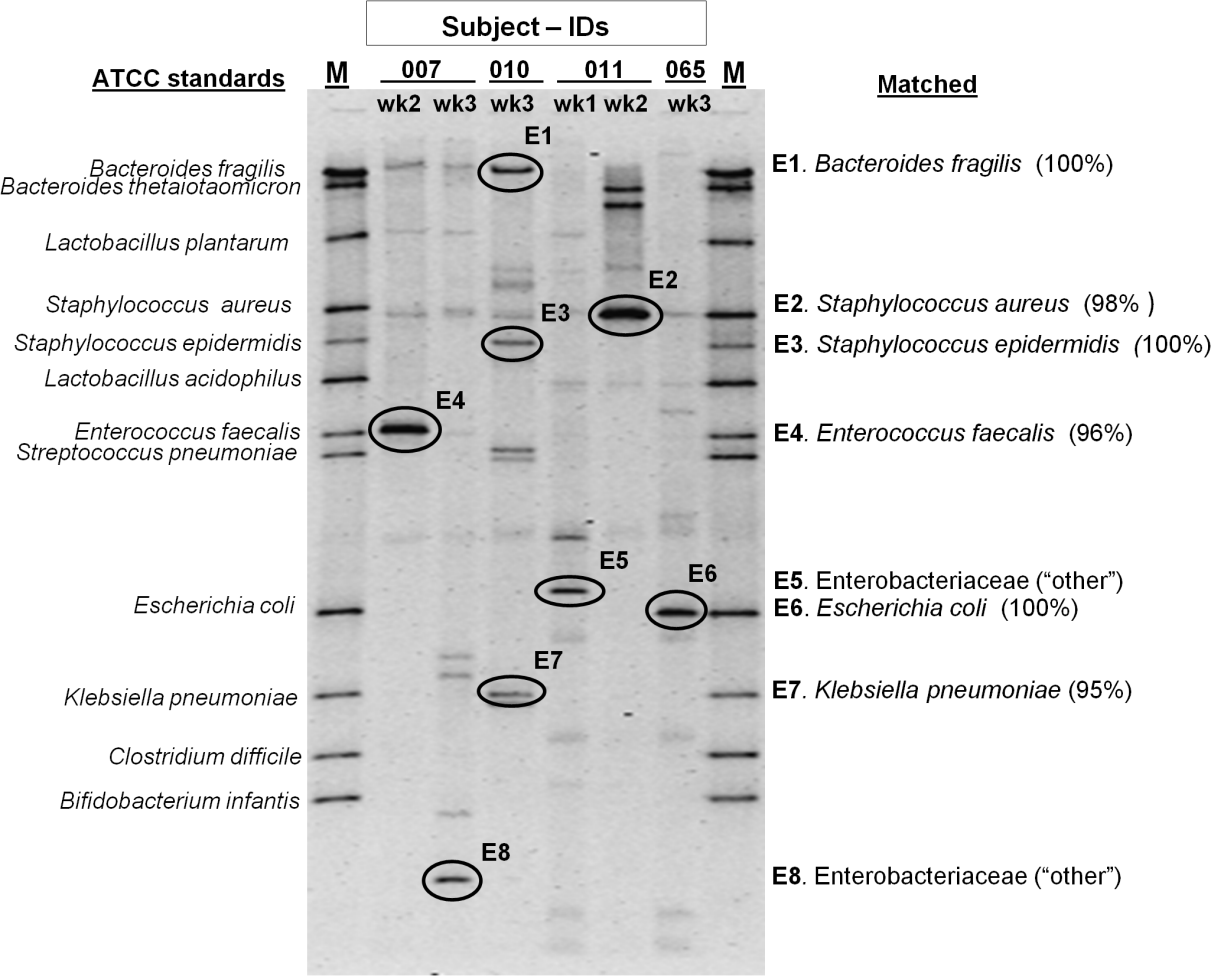
Elution of DGGE bands for sequence analysis. Identification of bacterial species based on BLAST alignment (% similarity) to 16S rRNA NCBI database. Bacteria listed as “matched” showed identical DGGE band migration pattern in line with respective ATCC standards (known DNA marker). Bands not aligned with ATCC standards showed sequence homology to other Enterobacteriaceae species in the NCBI RDP database.

For precise matching of bacterial species specific PCR amplicons, a DGGE marker was developed by co-amplification of standard ATCC genomic DNAs that included a panel of common aerobic/anaerobic bacterial species described earlier in preterm infant stools [5]. All ATCC strain DNA amplified 16S rDNA V3-amplicons (~200bp), and resulted in a sequence specific band resolution in DGGE (35% to 55% gradient gel), forming a DNA ladder (Marker lane, Fig 1) in line with- *Bacteroides thetaiotaomicron* (ATCC 29148D-5), *Bacteroides fragilis* (ATCC 25285D), *Lactobacillus plantarum* (ATCC BAA-793D-5), *Staphylococcus aureus* (ATCC 33591D-5), *Staphylococcus epidermidis* (ATCC 12228D-5), *Lactobacillus acidophilus* (ATCC 4357D-5), *Enterococcus faecalis* (ATCC 700802D-5), *Streptococcus pneumoniae* (ATCC 6314D-5), *Escherichia coli* (ATCC 35638D-5), *Klebsiella pneumoniae* (ATCC BAA-1706D-5), *Clostridium difficile* (ATCCBAA-1382D-5), and *Bifidobacterium infantis* (ATCC 15697D-5). The unmatched DGGE bands in GA samples (out of coverage of DGGE ladder), were scored as unknown bacterial species for diversity comparisons. DGGE was performed using a Biorad DCode Universal Mutation Detection System (Bio-Rad, Hercules, CA). The PCR products were resolved on 8% polyacrylamide (acryl amide/bisacrylamide, 37.5:1) gels in 0.5x TAE buffer (20 mM Tris-base pH-7.4, 10 mM Sodium acetate and 0.5 mM Na2EDTA). The denaturing gradient was prepared by a Gradient former (model 485 Bio-Rad) with 35% and 55% denaturant stock solutions. A 100% denaturant is defined as 7M Urea and 40% de-ionized formamide. Gel electrophoreses was performed at 60V for 14 hours at 60°C. Gels were stained with SYBR-Green DNA stain for one hour with gentle shaking, and digitized under UV fluorescence. The identical sized PCR products (191bp) from the V3-region, co-amplified uniformly in all sample DNA, produced informative DGGE profiles permitting identification of the common bacterial species that matched with our DGGE standard prepared from a panel of known bacterial species, under identical PCR-DGGE setup.

The similarity between DGGE profiles were visualized under UV illumination and digitized using a Gel-Doc 2000 RS-170/CCIR (Software BIORAD, Quantity one-4.4.1 1998, BIORAD laboratories Inc., USA). DGGE profiles were assessed based on presence or absence of individual bacterial species/bands in samples that matched with known standards. Qualitative analysis of bacterial species helped pattern correlations of bacteria colonizing the upper GI tracts during first 4 weeks of life. Overall, diversity comparisons also included a several unidentified DGGE bands (out of coverage of the DGGE standard utilized in this study).

### Elution of prominent DGGE bands for sequence analysis

A total of ten average intensity DNA bands (from unstained lanes matching with stained lanes) representing predominant bacteria in GA samples were excised from the DGGE gel using a sterile scalpel blade, suspended in gel extraction buffer and processed per manufacturer’s gel-elution protocol (Cat.#28704, Qiagen Inc.). The purified DNA from both known and unknown bands in DGGE were sequenced using the reverse primer (518R, without the GC-clamp) by BigDye Terminator Cycle sequencing kit (Applied Biosystems, CA), run in an automated sequencer (ABI 3730 DNA Analyzer) at the University of Nebraska Medical Centers’ (UNMC, Omaha, NE) Genomics core facility. The eluted DNA was also reamplified using V3-primers (with GC-clamp) and resolved to verify the position of the original DGGE band. The resulting DNA sequences (after removing the adjacent 40-base GC clamp) were submitted for phylogenetic analysis in the BLAST/NCBI database (http://www.ncbi.nlm.nih.gov/BLAST) to determine their closest 16S rRNA-V3 sequence based bacterial identity. Results of two bands were excluded from analysis due to the presence of mixed sequences.

### PCR screening of GA samples

GA samples from all neonates at all four time points were subjected to PCR for confirmation of presence or absence of major bacterial genera/species identified by DGGE. Primers, gene targets, and references are given in Table 1 [25–34].

**Table 1:** Primers and gene targets used for confirmation of common bacterial species identified in DGGE profiles.

Bacteria (spp.)	Primer Sequence (5’-3’)	A^Temp^ (°C)	Amplicon (bp)	Target	Primer Reference
*Bacteroides spp*.	F- GAGAGGAAGGTCCCCCAC	59	106	16S	Layton et al. 2006 [25]
	R- CGCTACTTGGCTGGTTCAG				
*Bifidobacteria spp*.	F- GCGTGCTTAACACATGCAAGTC	59	126	16S	Penders et al. 2005 [26]
	R- CAC CCGTTTCCAGGAGCTATT				
*Escherichia coli*	F- CATGCCGCGTGTATGAAGAA	59	96	16S	Huijsdens et al. 2002 [27]
	R- CGGGTAACGTCAATGAGCAAA				
*Lactobacillus spp*.	F- TGGATGCCTTGGCACTAGGA	58	92	16S	Haarman & Knol. 2006 [28]
	R- AAATCTCCGGATCAAAGCTTACTTAT				
*Clostridia spp*. *(cluster IV)*	F- GCACAAGCAGTGGAGT	60	239	16S	Matsuki et al. 2004 [29]
	R- CTTCCTCCGTTTTGTCAA				
*Enterococcus spp*.	F- AGAAATTCCAAACGAACTTG	55	91	23S	Frahm & Obst.2003 [30]
	R- CAGTGCTCTACCTCCATCATT				
*Klebsiella pneumoniae*	F- ATTTGAAGAGGTTGCAAACGAT	54	130	16S-23S	Liu et al. 2008 [31]
	R- TTCACTCTGAAGTTTTCTTGTGTTC				
*Staphylococcus aureus*	F- TCGGTACACGATATTCTTCAC	52	179	Sa442	Tan et al. 2001 [32]
	R- ACTCTCGTATGACCAGCTTC				
*Helicobacter pylori*	F- GGATAAGCTTTTAGGGGTGTTAGGGG	58	140	glmM	Espinoza et al. 2011 [33]
	R- GCATTCACAAACTTATCCCCAATC				
*Ureaplasma spp*.	F- CCTGCTTCGTTTAATGTATCTG	55	158	UreC	Cunningham et al. 2013 [34]
	R- GAAGATCCAATCTTTGAACAAATCGTA				

### Statistical analysis

The demographic data, covariates, and results from DGGE gels were scored in Microsoft Excel-2010 worksheet, and the data were analyzed using statistical analysis software SAS 9.3 version (SAS Institute Inc., Cary, NC, USA). Wilcoxon signed rank test or Kruskal-Wallis were performed when appropriate, to determine the association of multiple maternal and infant variables with total number of bacterial species, anaerobes and aerobes from first through fourth week of life. Maternal variables included membrane rupture, maternal bacteremia, placental pathology (chorioamnionitis), group B *Streptococcus* status, and intra-partum antibiotics. Neonatal variables included gender, race, place of birth (born in/outside of study hospital), mode of delivery, respiratory distress syndrome, patent ductus arteriosus, assisted ventilation, post-partum steroids, H-2 blockers, duration of antibiotics, sepsis status, and NEC. Spearman’s rank correlation was used to examine the relationship between gestational age, birth weight, and total number of bacterial species, anaerobes and aerobes from first through fourth week of life. Patterns of individual bacterial species colonization were examined using χ^2^ tests or Fisher’s exact test when appropriate. Trends for total number of bacteria, anaerobes and aerobes were compared for EBM and PBM fed neonates, using mixed model repeated measures with autoregressive covariance matrix and post-hoc Bonferroni correction at level of significance 0.05.

## Results

All neonates in our NICU were fed with either exclusive breast milk (EBM) or partially breast milk and preterm formula (PBM), via intermittent gavage-feeding. Eleven preterm neonates received EBM and eleven preterm neonates received PBM feeding in the first month of life. Table 2 shows the demographic and clinical characteristics of our study population. We found no statistically significant differences in demographic and clinical characteristics of EBM and PBM fed neonates.

**Table 2:** Demographic and clinical characteristics of preterm neonates in the NICU setting.

Demographic and clinical characteristics		EBM fed neonates^†^ (N = 11) (%)	PBM fed neonates^††^ (N = 11) (%)	Total neonates (N = 22) (%)
Gender	Male	4 (36.4)	5 (45.5)	9 (40.9)
	Female	7 (63.6)	6 (54.6)	13 (59.1)
Race	Black	5 (45.5)	9 (81.8)	14 (63.6)
	Non-black	6 (54.6)	2 (18.2)	8 (36.4)
Gestational age (weeks)*		26.87 (±2.18)	28.48 (±3.28)	27.68 (±2.84)
Birth weight (grams)*		980.91 (±274.58)	965.45 (±332.93)	973.18 (±297.9)
Low birth weight groups	Very low birth weight (<1500 - ≥ 1000 grams)	6 (54.5)	5 (45.5)	11 (50)
	Extremely low birth weight (<1000 grams)	5 (45.5)	6 (54.5)	11 (50)
Event	Outborn	2 (18.2)	1 (9.1)	3 (13.6)
	Inborn	9 (81.8)	10 (90.9)	19 (83.4)
Mode of Delivery	Normal vaginal delivery	7 (63.6)	3 (27.3)	10 (45.5)
	Caesarean section	4 (36.4)	8 (72.7)	12 (54.6)
Rupture of Membrane	≤4 hours	5 (45.5)	6 (54.6)	11 (50)
	>4 hours	6 (54.5)	5 (45.5)	11 (50)
Respiratory distress		11 (100)	10 (90.9)	21 (95.5)
Patent ductus arteriosus		3 (27.3)	3 (27.3)	6 (27.3)
Chronic lung disease		6 (54.5)	7 (63.6)	13 (59.1)
Sepsis	Clinically suspected	9 (81.8)	8 (72.7)	17 (77.3)
	Laboratory confirmed	2 (18.2)	3 (27.3)	5 (22.7)
Necrotizing Enterocolitis		0	1 (9.1)	1 (4.5)
Post-partum steroids		3 (27.3)	4 (36.4)	7 (31.8)
H-2 blockers		0	3 (27.3)	313.6)
Assisted ventilation		10 (90.9)	9 (81.8)	19 (86.4)
Antibiotic duration	≤72 hours	4 (36.4)	4 (36.4)	8 (36.4)
	>72 hours & ≤7 days	3 (27.3)	4 (36.4)	7 (31.8)
	>7 days	4 (36.4)	3 (27.3)	7 (31.8)
Intrapartum antibiotics	Yes	8 (72.7)	4 (36.4)	12 (54.6)
	No	3 (27.3)	7 (63.6)	10 (45.5)
Chorioamnionitis	Yes	0	1 (9.1)	1 (4.5)
	No	7 (63.6)	4 (36.4)	11 (50)
	Unknown	4 (36.4)	6 (54.5)	10 (45.5)
Maternal bacteremia	Yes	1 (9.1)	1 (9.1)	2(9.1)
	No	1 (9.1)	1 (9.1)	2 (9.1)
	Unknown	9 (81.8)	9 (81.8)	18 (81.8)
Maternal group B Streptococci	Yes	2 (18.2)	2 (18.2)	4 (18.2)
	No	1 (9.1)	1 (9.1)	2 (9.1)
	Unknown	8 (72.7)	8 (72.7)	18 (72.7)

^†^EBM: exclusively breast milk fed neonate.

^††^PBM: partially breast milk fed (breast milk + preterm formula) neonate.

*Values are mean (standard deviation).

Identification and enumeration of bacterial species were done by DGGE using 12 ATCC purified DNA bands as markers. For further confirmation of species, eight DGGE bands from several GA samples were eluted (Fig 1) and sequenced. Six of these showed high DNA homology with the respective bacterial species by BLAST. Two bands (E5 and E8) showed homology to multiple species (Shigella, Salmonella, Enterobacter) and hence, were labeled as Enterobacteriaceae (Table 3). These bands were counted under “other” category for our analyses. PCR results of all samples for the eight common bacterial species were in agreement with the DGGE results. With the exception of PCR negativity for Klebsiella, Lactobacillus, and Bifidobacteria in a very small number of samples, almost all samples were PCR positive for the specific bacterial species when they were recorded in DGGE. We also examined all our samples for *H*. *pylori* and *Ureaplasma*. Both were negative in all time points for all 22 neonates.

**Table 3:** Sequence analysis of bands shown in Fig 1.

Band eluted (E1-E8)	BLAST (% Similarity)	*Nearest species ID	ATCC Marker	V3-16S rRNA, partial sequence
E1	100%	* Bacteroides fragilis*	Matched	TATTCTTATATAAAAGAAGTTTACGACCCATAGAGCCTTCATCCTTCACGCTACTTGGCTGGTTCAGGCTAGCGCCCATTGACCAATATTCCTCACTGCTGCCTCCCGTA
E2	98%	*Staphylococcus aureus*	Matched	TTCTTCCCTAATAACRGAGTTTTRCGATCCGAAGACCTTCATCACTCACGCGGCGTTGCTCCGTCAGGCTTTCGCCCATTGCGGAAGATTCCCTACTGCTGCCTCCCGTA
E3	100%	*Staphylococcus epidermidis*	Matched	TTCTTCCCTAATAACAGAGTTTTACGATCCGAAGACCTTCATCACTCACGCGGCGTTGCTCCGTCAGGCTTTCGCCCATTGCGGAAGATTCCCTACTGCTGCCTCCCGTA
E4	96%	*Enterococcus faecalis*	Matched	TTCTTCTCKAAYAACAGAGTTTTACGATCCGAAAACCTTCTTCACTCACGCGGCGTTGCTCGGTCAGRCTTTCGYCCATTGCCGAAGATTCCCTACTGCTGCCTCCCGTA
E5	96%	Enterobacteriaceae (“other”)	New	TKCCKCCCCGCTGAAAGTACKTTRCAACCCGAAGGCCTTSWTCATACACGCGGCATGGCTGCATCAGGCTTGCGCCCATTGTGCAATATTCCCCACTGCTGCCTCCCGTA
E6	100%	*Escherichia coli*	Matched	TTCCTCCCCGCTGAAAGTACTTTACAACCCGAAGGCCTTCTTCATACACGCGGCATGGCTGCATCAGGCTTGCGCCCATTGTGCAATATTCCCCACTGCTGCCTCCCGTA
E7	95%	*Klebsiella pneumoniae*	New	MTTYCYTYCCGMTGAAGKRCTTTMCAACCCGAAGGCCTTCTTCAYMCACGCGGYATGGCTGSATCAGGCTTGCGCCCATTGTGCAATATTCCCCACTGCTGCCTCCCGTA
E8	100%	Enterobacteriaceae (“other”)	New	CTTCCTYCCCGMYGAAKTACTTMACAACCMGAAGGCCTTCTTCATACACGCGGMATGGCTGYATCAGGCTTGCSCCCATTGTGCAATATTCCCCACTGCTGCCTCCCGTA

*Due to high sequence homology shared by different bacterial species in the 16S-V3 region band E5 and E8 showed similarity with other members of the family Enterobacteriaceae.

### Identification and enumeration of bacterial species

**Fig 2:**
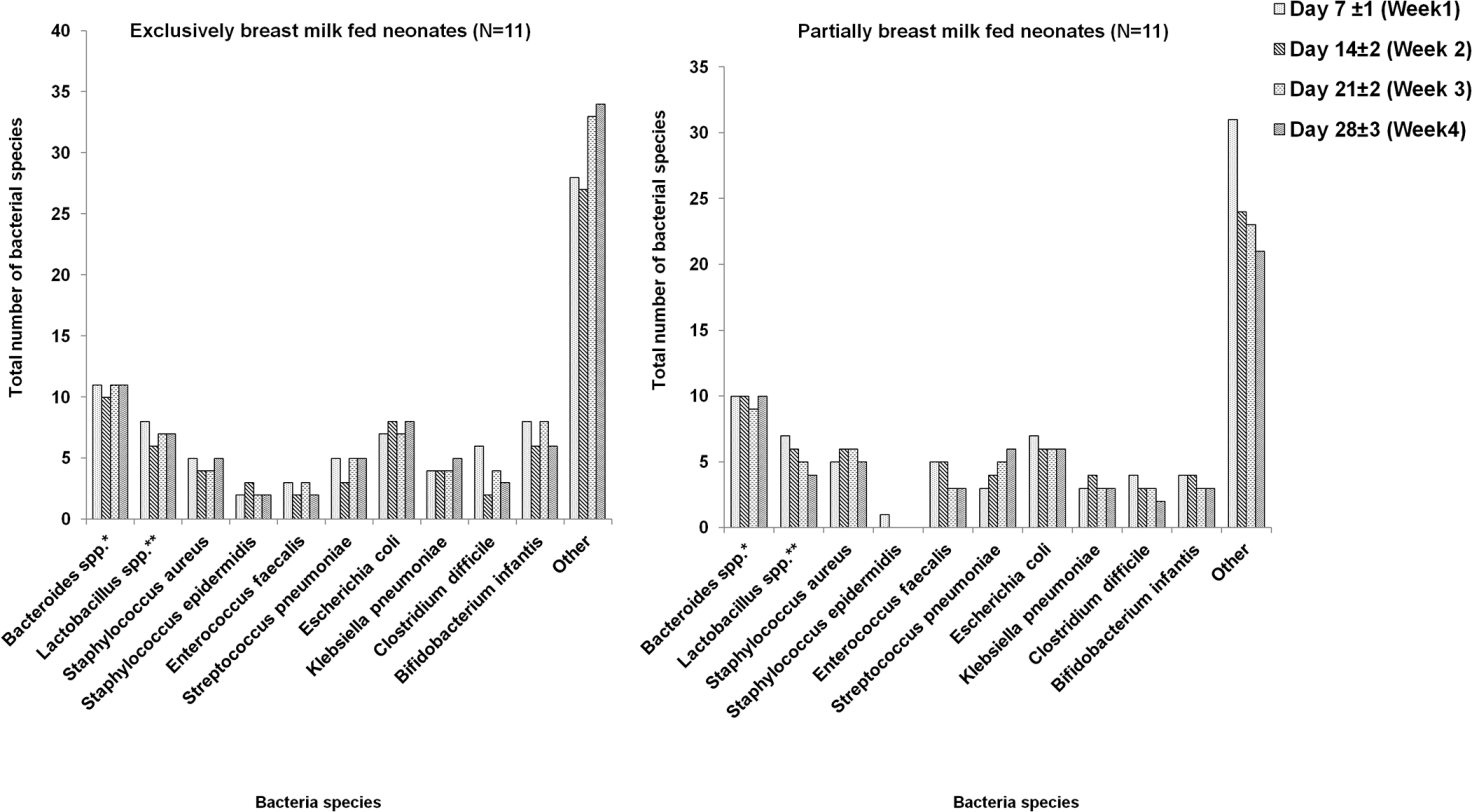
Individual bacterial species detected in gastric aspirate samples of preterm neonates during first four weeks. Number of bacterial species detected in gastric aspirate samples of preterm neonates during the first four weeks of life, by feeding type. The X-axis in each graph represents individual bacterial species, and the Y-axis in each graph represents the total number of bacterial species. Partially breast milk fed infants were fed breast milk and preterm formula. *Bacteroides spp. includes *Bacteroides fragilis* and *Bacteroides thetaiotaomicron;* **Lactobacillus spp. includes *Lactobacillus plantarum* and *Lactobacillus acidophilus*. Other includes DGGE bands that did not correspond to the ATCC standards in the marker. Bacterial species were counted based on DGGE bands.

Individual bacterial species isolated from gastric aspirates of all neonates in our study over the first four weeks of life are given in Fig 2. While there was similarity between a few time points (i.e., week one through four) in any individual neonate and some bacteria were common among infants, there were distinct differences among the 22 neonates studied. Even within the four week period, appearance and disappearance of many predominant DGGE bands clearly pointed toward the unstable and changing nature of gastric colonization by known and several unidentified bacterial species. All neonates (with the exception of a single neonate in the PBM group at week-one) in their first month of life, regardless of the feeding type, were uniformly colonized by *Bacteroides spp*. Other commonly identified bacteria in the first month of life were *E*. *coli*, *Lactobacillus spp*., *B*. *infantis*, *S*.*aureus*, and *C*.*difficile*. Compared to the first week, there appeared to be an overall decline in *B*. *infantis* colonization in the fourth week of life which was not statistically significant. However, EBM-fed neonates had higher colonization of *B*.*infantis* in the first week (p = 0.03) and third week (p = 0.03) of life compared to PBM-fed neonates. This difference in both EBM-fed and PBM-fed neonates diminished, and was not significant by the end of the fourth week. After adjusting for feeding type, we observed no significant trend in any bacterial species identified (other than *Bacteroides spp*. and *B*.*infantis*) by the end of the fourth week of life. We did not detect *Ureaplasma spp*. or *H*. *pylori* in any of our samples either by DGGE or PCR.

#### Total number of bacterial species

We examined for changes in total number of bacterial species colonized in gastric samples over the four week study period for all neonates, with and without adjusting for feeding type. The time-trend analysis showed a decrease in average total number of bacterial species from first week (median 7; inter-quartile range (IQR) 1 vs. median 9; IQR 6) to fourth week (median 5; IQR 3 vs. median 7; IQR 3) in PBM-fed neonates compared to EBM-fed neonates (Kruskal Wallis, p = 0.05). We examined for influence of various neonatal and maternal demographic and clinical characteristics on total bacterial species from first through fourth week of life. Univariate analyses suggested that neonates born to mothers with membrane rupture of duration > 4 hours (median 9; IQR 1) had higher total number of bacterial species than neonates born to mothers with membrane rupture ≤ 4 hours (median 7; IQR 4) in the first week of life (Kruskal-Wallis p = 0.05). However, this difference was not observed at week two, three or four. Birth weight had a positive correlation with number of bacterial species colonized in PBM babies only at week-4 (Spearman’s rho = 0.83, p = 0.002) with no impact during weeks 1–3. None of the other maternal or neonatal characteristics showed an association with total number of bacterial species colonizing in first through fourth week.

For further analysis by group of bacteria, we categorized *Bacteroides spp*., *B*.*infantis*, *Lactobacillus spp*., *E*. *faecalis* and *C*. *difficile* as anaerobes, and *S*. *aureus*, *S*. *epidermidis*, *S*. *pneumoniae*, *E*. *coli* and *K*. *pneumoniae* as aerobes.

#### Anaerobic bacterial species

*Bacteroides spp*. was included in the total number of anaerobic species colonized. However trend analysis was not performed for *Bacteroides spp*., since it was consistently present in all neonates over the four week period (Fig 2). The number of anaerobic species in the first week of life was significantly higher in EBM-fed neonates compared to PBM-fed neonates (median 3; IQR 1 vs median 2; IQR 1, Kruskal-Wallis p = 0.04). Among anaerobes, we observed an early colonization by Bifidobacteria (>50%) during the first 3 weeks that reduced to 36% by the fourth week. After adjusting for feeding type, the number of anaerobes, showed a significant decreasing trend over time (mixed model repeated measures ANOVA p = 0.03). Bifidobacteria colonization was higher in EBM-fed neonates (nearly 80–90%) than in PBM-fed neonates (nearly 18–27%) until the third week of life. By the end of the fourth week, all neonates in NICU had lower incidence of Bifidobacteria, regardless of their feeding type. *E*.*faecalis*, *C*.*difficile*, *Lactobacillus spp*. colonization showed no significant trend over time with/without adjusting for feeding type.

Spearman’s correlation analysis revealed no significant correlation between gestational age, birth weight and anaerobic colonization from first through fourth week of life. However, birth weight was associated with anaerobic colonization only during the fourth week of life in PBM-fed neonates (Spearman’s rho = 0.79, p = 0.004). After adjusting for feeding type, the correlation between birth weight and number of anaerobic species did not exist.

In the current study, univariate analysis showed that colonization by anaerobes was significantly higher in outborn or transfer cases (median 5; IQR 1) compared to those born in our study hospital (median 3; IQR 1) in the first week of life (Kruskal-Wallis p = 0.006). Outborn neonates showed higher incidence anaerobes such as *Bifidobacterium spp*. (100%), *E*.*faecalis* (100%), *C*. *difficile* (100%), and *Lactobacillus spp*. (100%) compared to inborn neonates that showed *B*. *infantis* (47.4%), *E*. *faecalis* (31.6%), *C*. *difficile* (36.8%), and *Lactobacillus spp*. (47.4%) in the first week. Again, this difference was not evident at weeks 2–4. Except place of birth (inborn/outborn), other maternal and neonate variables under study showed no significant change in colonization patterns for anaerobic bacterial species over time (weeks1-4).

#### Aerobic bacterial species

*E*.*coli and S*.*aureus* showed higher prevalence compared to *K*.*pneumoniae*, *S*.*pneumoniae* and *S*.*epidermidis* from first to fourth week (Fig 2). We observed unstable and dynamic colonization pattern for all aerobes in GA samples of preterm NICU neonates. There was no significant trend observed over the four week period in EBM and PBM-fed neonates. Due to frequent variations in the acquisition of aerobic microbiota in the stomach, no significant relationship was observed between these colonization by species and maternal and neonatal variables.

### Diversity among neonates and gastric colonization in neonatal necrotizing enterocolitis (NEC)

**Fig 3:**
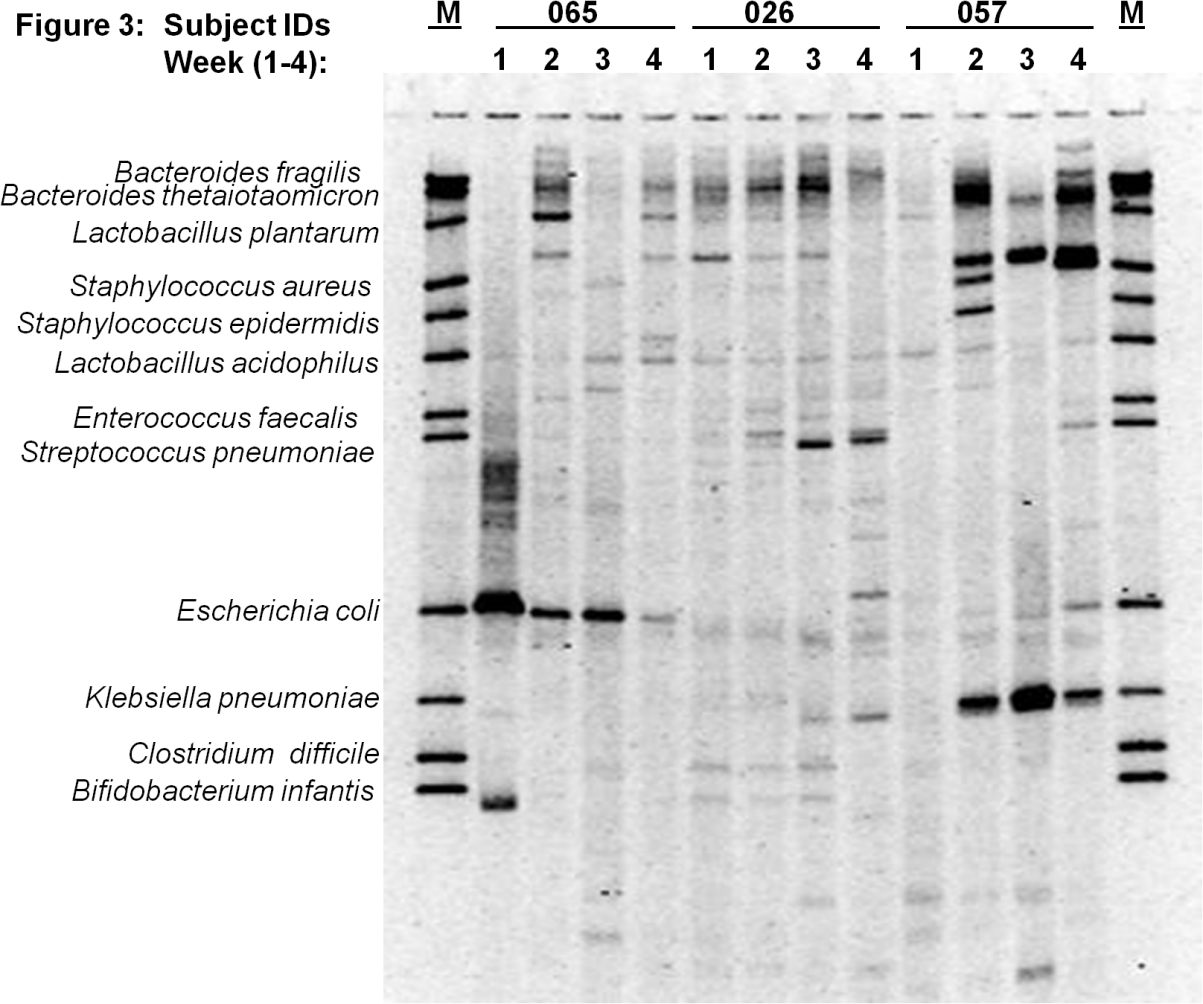
Microbial diversity in the developing neonatal stomach for study Ids 065, 026, 057. The gel figure shows the DGGE profiles of gastric aspirates from preterm infants (Id 065, 026 and 057) collected during week 1 through 4. Each figure shows the GA profiles of three neonates at four time points. Note the overall diversity among neonates and within neonates during the four week period. GA pattern of neonate # 57 with NEC exhibits strong colonization with *E*. *coli*, and lack of species diversity.

**Fig 4:**
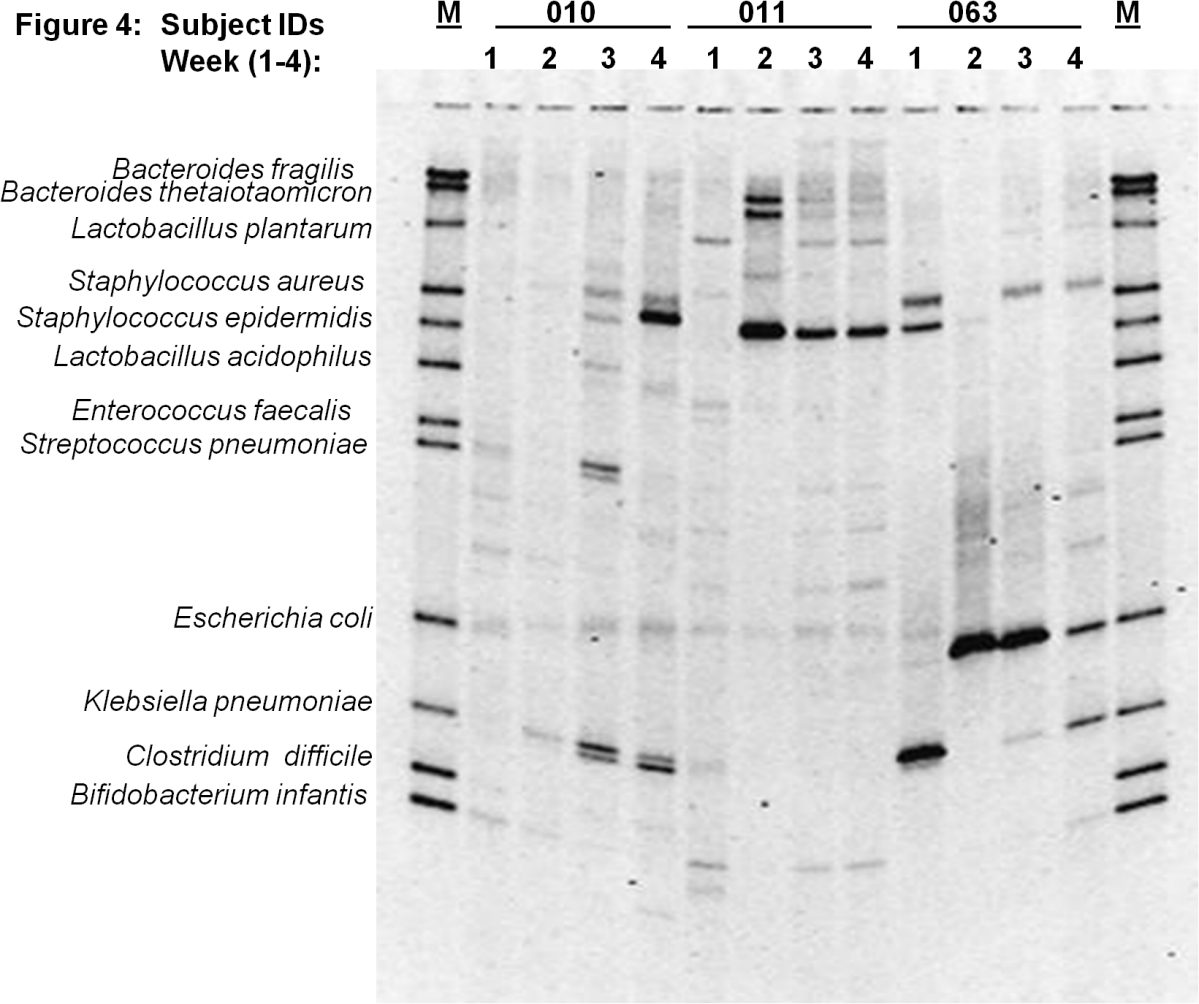
Microbial diversity in the developing neonatal stomach for study Ids 010, 011, 063. The gel figure shows the DGGE profiles of gastric aspirates from preterm infants (Id # 010, 011 and 063) collected during week 1 through 4. Each figure shows the GA profiles of three neonates at four time points. Overall paucity of bacteria is evident in gastric aspirates from Ids 010 and 63.

**Fig 5:**
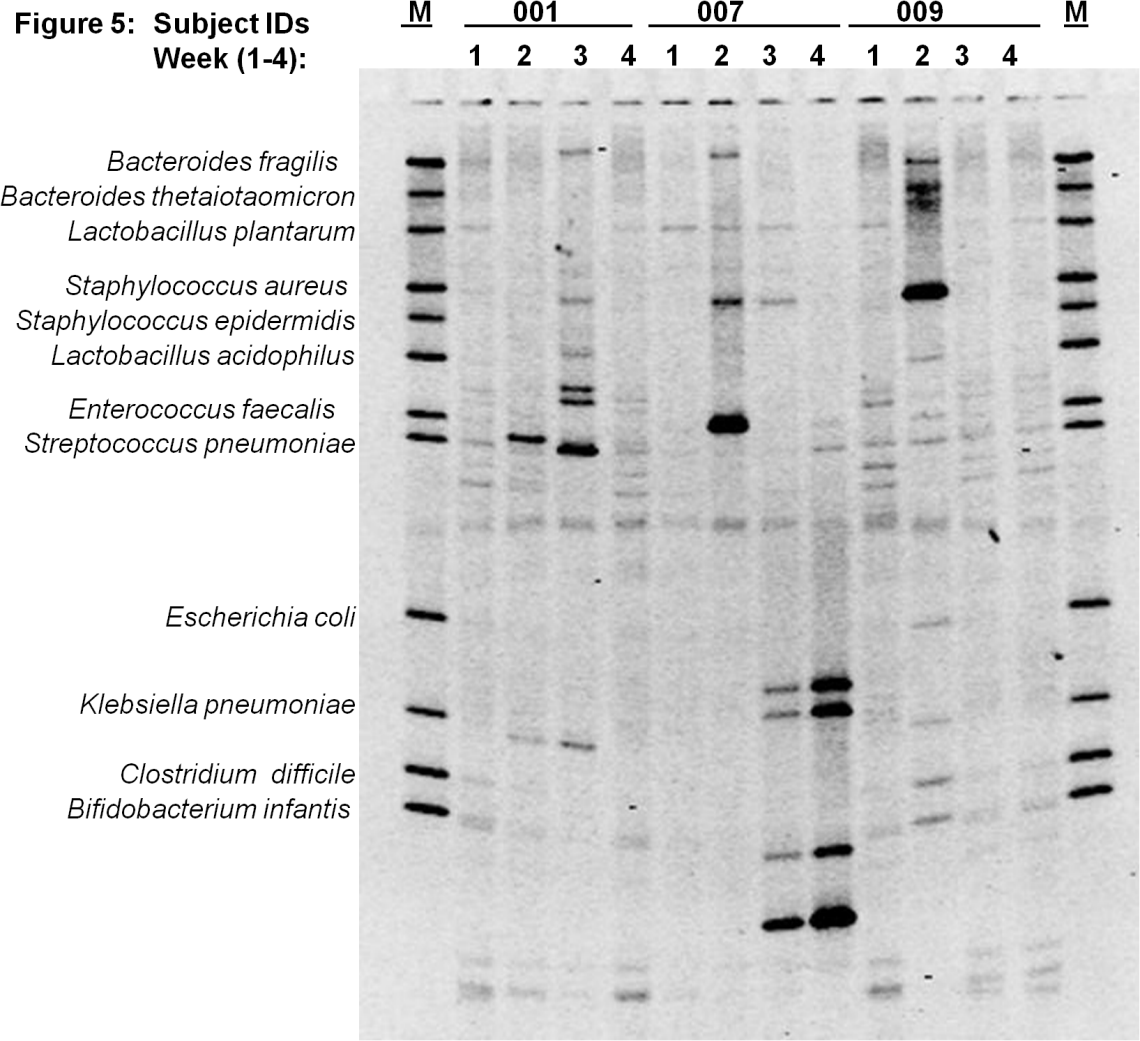
Microbial diversity in the developing neonatal stomach for study Ids 001, 007, 009. The gel figure shows the DGGE profiles of gastric aspirates from preterm infants (Id 001, 007 and 009) collected during week 1 through 4. Each figure shows the GA profiles of three neonates at four time points. Overall paucity of bacteria is observed in gastric aspirates from Id 001 compared to Ids 007 and 009.

**Fig 6:**
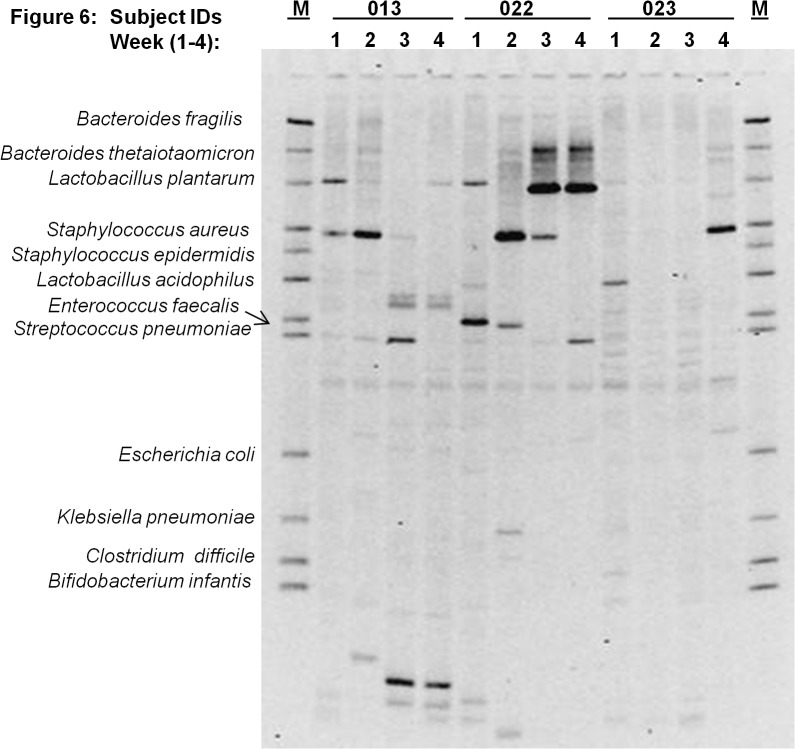
Microbial diversity in the developing neonatal stomach for study Ids 013, 022, 023. The gel figure shows the DGGE profiles of gastric aspirates from preterm infants (Id 013, 022 and 023) collected during week 1 through 4. Each figure shows the GA profiles of three neonates at four time points. Overall paucity of bacteria was observed in gastric aspirates from Ids 013 and 023.

Fig 3 through 7 describe the weekly colonization patterns of fifteen preterm neonates in our study. Samples run in a set of four for each neonate from week one through four in these gels demonstrate the unstable and dynamic nature of colonization in our preterm neonate population. However, it should be noted that some bands were present at two or more time points in several neonates. This includes Klebsiella and *S*. *aureus* in week 2, 3, and 4 in neonate # 57 (Fig 3), *E*. *coli* in week 2 and 3 in neonate # 63 (Fig 4), *S*. *epidermidis* at all four time points in neonate # 11 (Fig 4), Klebsiella and *S*. *aureus* and *S*. *epidermidis* in week 3 and 4 (Fig 4), *L*. *plantarum* in week 1 and 2 in neonate #7 (Fig 5) and *L*. *plantarum* in week 3 and 4 in neonate # 22 (Fig 6). While these patterns point toward commonality of a handful of bacteria but not the rest (suggesting attempt of certain species to remain stable), it is intriguing to note that in some neonates such as neonate # 23 (Fig 6) and neonate # 9 (Fig 5) where each time points exhibited a different colonization pattern. While we could not attribute any biological significance to these types of colonization in most of our neonates, one neonate (# 65, Fig 3) in our study developed clinical necrotizing enterocolitis (NEC) on day six of life. This neonate was a black female, born via spontaneous vaginal delivery at 25.1 weeks of gestation, weighed 715 grams at birth with Apgar scores 6, 8 at 5 and 10 minutes. The neonates’ mother received intra-partum antibiotics and steroids, and presented with history of amniotic membrane rupture 6 days and 18 hours. The neonate received early antibiotics for >7 days, and was initially fed with preterm formula in first week followed by PBM in later weeks of life. At birth, the neonate suffered from respiratory distress and received post-partum steroids, assisted ventilation, and was diagnosed with chronic lung disease on day-28 of life. Fig 3 (neonate # 65, lanes 1–4) shows the DGGE profile of the neonate with NEC during the first four weeks of life. The NEC neonate showed reduced number of total bacteria species (faint and fewer number of total DGGE bands) compared to neonates without NEC. The neonate showed a total lack of Bifidobacteria, however, *E*.*coli* was consistently identified during all 4 weeks, showing very strong bands in week 1 through 3. Among anaerobes, *Bacteroides spp*., *Lactobacilli spp*., and *E*. *faecalis* appeared only in gastric samples of week 2 and 4. Several unidentified bands persisted in first month of life for this neonate.

**Fig 7:**
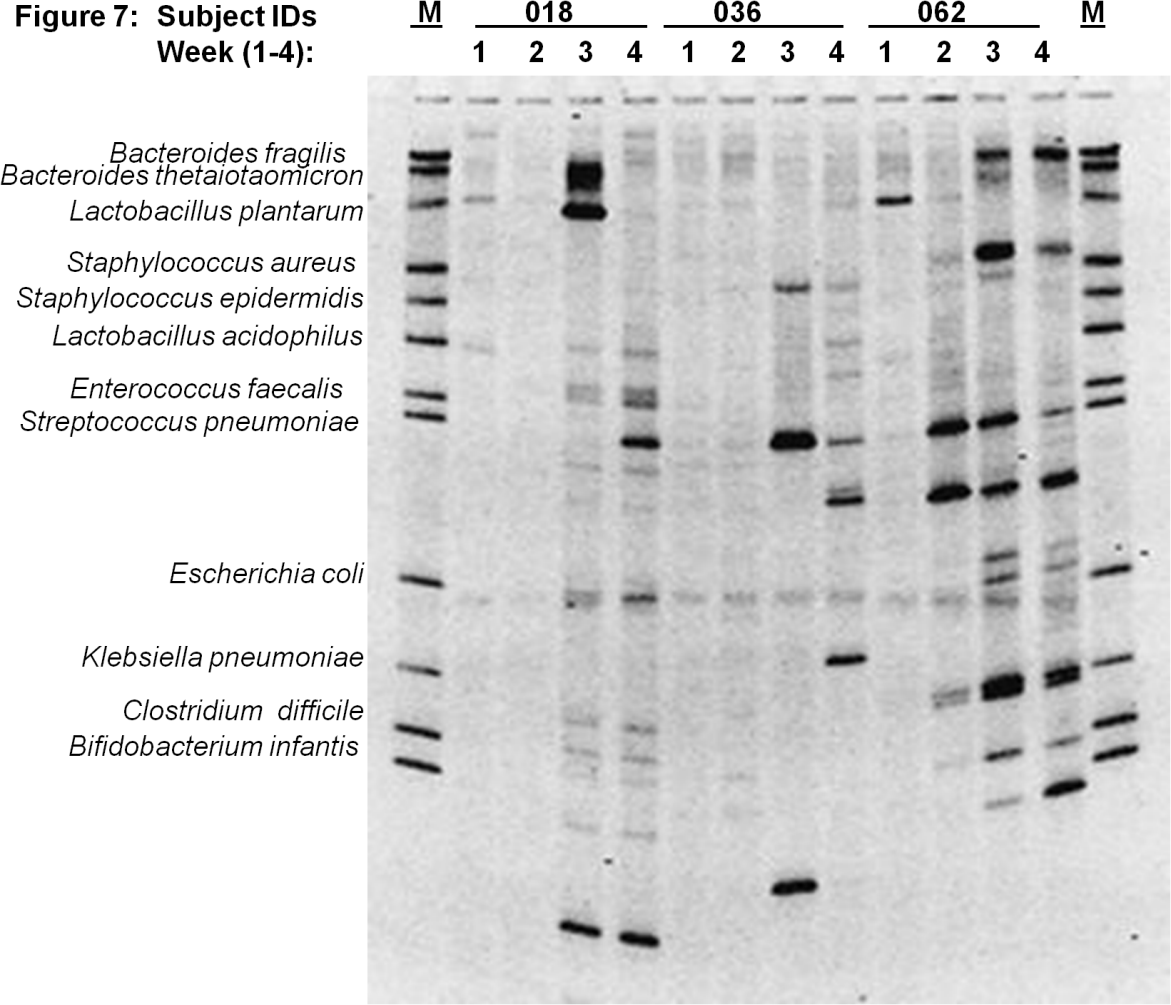
Microbial diversity in the developing neonatal stomach for study Ids 018, 036, 062. The gel figure shows the DGGE profiles of gastric aspirates from preterm infants (Id 018, 036 and 062) collected during week 1 through 4. Each figure shows the GA profiles of three neonates at four time points.

## Discussion

In the present study, overall PCR-DGGE of gastric samples obtained from 22 preterm neonates in their first 4 weeks of life showed a highly dynamic and unstable pattern of bacterial microbiota in the GA samples. Also, the number of bacterial species acquired was moderately low. Previous research focusing effect of different antimicrobial regimens on gastrointestinal (GI) tract microbiota in neonates indicates that prolonged antibiotic therapy and delayed feeding were associated with reduced number of bacterial species identified in fecal samples [2]. Although we do not have data from full term neonates on bacterial diversity, early exposure to antibiotics and relatively sterile NICU environment can be considered possible explanations for low to moderate number of bacterial species in our gastric samples. However, the diversity and types observed in gastric aspirates of our study population is in contrast to the microbiota of oral cavity in preterm neonates that showed a total lack of anaerobes [23]. These types of initial colonization may affect the cascade of distal GI tract colonization leading to extensive GI tract diversity. Evidences suggest that lack of microbial diversity early in life may predispose to pathologic conditions such as gastrointestinal tract inflammation and NEC [14,22]. Since initial colonization of the upper GI tract is expected to provide “seeding” for the lower GI tract, early acquisition and maintenance of specific microbiota in the stomach and upper intestine may be critical for shaping the GI microbiome of the neonate.

In our study, neonates showed acquisition of *Bacteroides spp*. (in all gastric aspirates) and *E*.*coli* as predominant species that remained unaffected by antibiotics and other factors in the NICU. While other studies have shown presence of *Bacteroides*, detection of this species in almost 100% of our neonates is important, and points to the utility of molecular methods not used in other studies. Blakey et al. reported that *Bacteroides spp*. were predominant species followed by gram negative aerobic bacilli in gastric microbiota in preterm neonates using culture techniques. By 9–12 days, 54% of specimens yielded *Bacteroides spp*. and *S*. *epidermidis*, *S*. *aureus* were less frequent [1]. Another study using culture technique showed abundance of *E*.*coli* followed by presence *Staphylococcus albus*, *S*.*aureus*, *Streptococcus faecalis*, and alpha hemolytic *Streptococci* in stomach of preterm LBW neonates in NICU setting [35]. Recently, Milisavljevic et al. used 16S rRNA sequencing analysis of gastric aspirate of ELBW NICU neonates and showed predominance of *S*.*aureus* and *S*.*epidermidis* followed by *Streptococcus*, and *Ureaplasma*. The authors also found an increase in the percentage of gram negative species such as *E*.*coli*, *Neisseria*, *Haemophilus*, *K*. *pneumoniae*, and *Pseudomonas aeruginosa* from 9% to 50% from first to fourth week of life with overall low to moderate diversity in total number of bacteria species that is consistent with our findings [36]. All gastric aspirates in our study were negative for *H*. *pylori* and *Ureaplasma* specific DNA. Lack of *H*. *pylori* is not unexpected in our newborn population since there are remote chances of person to person transmission in the NICU setting in the U.S. [37–39]. It is also not unusual to observe the absence of *Ureaplasma spp*, since we had only one case of chorioamnionitis in our study population. Milisavljevic and colleagues justified acquisition of *Ureaplasma spp*. in three out of 12 infants during the first week of life from infected amniotic fluid due to premature membrane rupture and prolonged labor in their population [36].

Prematurity seriously impairs gastrointestinal tract colonization; gastrointestinal tract maturity (gestational age >34wks) is known to play a vital role specifically in effective bifidobacterial colonization. Hence, gestational age at birth can be a major determinant for delayed acquisition of *Bifidobacteria spp*. in preterm neonates [40]. Availability and survival of these key members of the healthy lower GI tract microbiota should not be ignored. It is interesting to note that none of the small number of studies in preterm neonates using either culture technique [1,19] or sequence analysis [20] of gastric aspirates has identified presence of anaerobes such as Bifidobacteria and Lactobacilli. In our study, we could detect Bifidobacteria colonization in early weeks of life which was significantly higher in exclusively breast milk fed neonates until the third week of life compared to those receiving PBM. This phenomenon, however, disappeared in the fourth week when both groups looked similar. This may justify the need for breast feeding over an extended period in early life to promote Bifidobacteria acquisition and ultimate colonization of the lower GI tract. Research has shown that paucity of Bifidobacteria during early gastrointestinal tract development may predispose preterm ELBW neonates to aberrant GI tract colonization patterns, thereby increasing susceptibility to infection and GI tract inflammation such as NEC. Hence, strategies for prevention of NEC and other infections during prematurity should seriously consider sustaining EBM and/or bifidobacterial supplementation during these critical weeks of life.

H2-blocker therapy in preterm infants to treat gastric acidity has been linked to higher incidence of NEC and a 7.6 fold increase in rate of sepsis [41]. This relationship could not be explored in our study as we had only one NEC infant that did not receive H2-blockers during the first four weeks of life. Preterm NEC neonates have shown severe lack of bacterial diversity in lower gastrointestinal tract microbiota [20] with presence of *E*. *faecalis*, coagulase-negative Staphylococci and other Enterobacteriaceae [42]. A study comparing microbial profile of gastric aspirates of NEC vs. non-NEC preterm low birth weight neonates using culture techniques showed higher frequencies of *K*. pneumoniae and other gram negative aerobes [43]. The only preterm ELBW NEC neonate in our study also showed similar findings of low total number of bacteria species with presence of *E*. *coli*, *E*. *faecalis*, and *S*. *aureus*. It is also intriguing to note that other preterm infants (subject ID 1, 13, 23) in our study population that did not develop NEC also had an extremely limited bacterial diversity, but, did not colonize with *E*. *coli* unlike the neonate with NEC (subject ID 65). Results of a single case of NEC in our population cannot be generalized, and absence of diversity in the stomach cannot be linked to lower diversity in colonic microbiota. However, this does add to the accumulating evidence in the literature on low bacterial diversity and lack of beneficial anaerobes in these preterm infants triggering infectious and inflammatory conditions. While most of the neonatal and maternal demographics collected in our study appear to have minimal or no association, we did observe several factors that do play a role with upper GI bacterial colonization in the first week of life. In our study, membrane rupture influenced total number of bacteria species colonized in first week; whereas transfer of infants from other hospitals influenced the total number of anaerobic species colonized in the first week of life. Brook et al. suggested similar correlation between birth weight and anaerobes [45], and exposure to outside world/fomites and handling by multiple persons clearly seem to influence the availability of multiple species including anaerobes and colonization by these species during early life [44].

Although our sample size was relatively small, we employed extensive statistical methods to examine quantitative changes of statistical differences as well as any changes in patterns. We used non-parametric Wilcoxon-signed rank or Kruskal-Wallis or Spearman’s rank correlation where appropriate to address the limitation of smaller sample size and skewed distributions. Such limitations restrict analysis of longitudinal data using repeated measure analysis of variance (ANOVA) influencing the statistical power of the study. A simulation study showed that Mixed Effects Model (MEM) has 80% power and higher efficiency when sample size is as small as 20 with four repeated measures compared to conventional repeated measures ANOVA [45]. Hence, use of mixed model for longitudinal data analysis is appropriate to address the limitations of normality and smaller sample size in our study. Longitudinal follow up of same infant over time for microbial profiles, introduces some random variation due to time and other uncontrolled factors such effect of medications, feeding, co-occurrence of bacterial species etc. We expect that the observations of week 1 and week 2 are more highly correlated than week 1 and week 4 due to time lag. To incorporate the random effect of time and covariance, we used autoregressive covariance matrix for a time-series analysis that assumes correlation of observation with previous observations considering the time lag between time points.

Once thought to be a sterile environment, the upper GI tract is now believed to be transiently colonized with limited bacterial species during birth and gastrointestinal tract development. This process can be vital for newborn health and immunity since primary bacterial acquisition may dictate the long-term establishment of a stable gastrointestinal tract microbiota with age. Distortions in intestinal microbiome have recently been shown to be associated with sepsis in premature infants [46]. In the same lines, Madan and colleagues have shown a predictive value of gastrointestinal tract microbiota in late onset neonatal sepsis, and proposed a role of protective bacterial species against this condition [47]. In the current study based on the PCR-DGGE bacterial profile in our population, prenatal and antenatal characteristics, we can conclude that gastric colonization starts in the very first week of life, and is highly dynamic. Although there is acquisition of new species over time, there is no linear growth in diversity during the first weeks in life as seen in the stool flora of preterm neonates [5]. Monitoring this colonization over a longer period of time may be required to conclusively comment on stabilization of upper GI microbiota during this critical period. We understand there is need for additional investigations in these lines to optimally utilize the information in clinical settings. For example, it is important to examine lower GI microbiota of infants in serial stool samples to identify concordance. Such studies will reveal if patterns initially identified in the upper GI microbiota can indeed be utilized in predicting lower GI colonization, and the timing and persistence of such colonization over a defined period of time. Further comparative studies on healthy term neonates and neonates with specific disease states will help us discern the difference among these infants. Depending on the specificity and sensitivity of such information, new tools and algorithms can be developed and utilized for early diagnosis and prognosis of GI conditions during neonatal and early infancy and other associated immunological changes that may be driven by gastrointestinal tract microbiota. Follow up over the longer term during childhood and beyond can reveal important information on how initial acquisition shapes late gastrointestinal tract microbiota and disease states. Results of such investigations, along with information on associated disease conditions may provide leads for early diagnosis of GI conditions, monitoring morbidity, and possibly design of therapeutic interventions and preventive modalities in the newborn period and beyond.

PCR-DGGE has the ability to discern single base sequence difference among species and resolve individual band profiles of predominant bacterial microbiota using 16S rRNA universal PCR-primers. We targeted the V3 region known to have the most sequence heterogeneity among bacterial species [48]. While we can confirm the presence of predominant bacterial species identified by sequence match and species-specific PCR, we have several bands that were pooled into an “other” category. At this point in time, although we do not have enough information to comment on these unidentified bands in our DGGE analysis, we want the readers to note that these bands or sequences may be of paramount importance in our pursuit of discovery. Studies in the literature now show that abundant phages and mobile elements in the ocean [49] as well as human gut [50,51]. In contrast to conventional wisdom that human microbiota of individuals are uniquely different from each other, a very recent report this month describes a 97 kbp sequence of a novel bacteriophage, crAssphage to be ubiquitous in the stool of persons around the world, which is also extremely abundant (90% and 22% of all reads in virus-like particle-derived metagenomes and total community metagenomes, respectively). It is in fact 1.68% of all human faecal metagenomic sequencing reads in the public databases. Till date this was not recognized by scientists, although it was present in public databases for quite some time. But, more importantly, authors of this report predict Bacteroides to be the host for this phage due to coding of Bacteroides-related protein homologues and a carbohydrate-binding domain unique to Bacteroides [52]. While Bacteroides are known to form a part of the healthy colonic flora in humans, our findings, for the first time, demonstrates the presence of this genus ubiquitously in most preterm neonates. It is possible that such upper intestinal colonization can serve as starter cultures in human neonates that may bear biological significance not known to us at present.
